# Social media use and everyday cognitive failure: investigating the fear of missing out and social networks use disorder relationship

**DOI:** 10.1186/s12888-023-05371-x

**Published:** 2023-11-24

**Authors:** Christian Montag, Sebastian Markett

**Affiliations:** 1 Department of Molecular Psychology, Institute of Psychology and Education, Ulm University, Helmholtzstr. 8/1, 89081 Ulm, Germany; 2 Molecular Psychology, Department of Psychology, Humboldt-Universität zu Berlin, Berlin, Germany

**Keywords:** Social media addiction, Social networks use disorder, Fear of missing out, Cognitive failure

## Abstract

**Background:**

Nearly five billion individuals worldwide are using social media platforms. While the benefits of using social media, such as fostering social connections, are clear, ongoing discussions are focused on whether excessive use of these platforms might have adverse effects on cognitive functioning. Excessive social media use shares similarities with addictive behaviors and is believed to result from a complex interplay of individual characteristics, emotions, thoughts, and actions. Among these contributing factors, one of particular interest is the Fear of Missing Out (FoMO), a state where an individual apprehends that others are experiencing rewarding moments in their absence (but see more information on the FoMO trait/state debate in this article).

**Methods:**

In this study, we aimed to explore the intricate relationships between FoMO, tendencies towards Social Networks Use Disorder (SNUD), and everyday cognitive failures. To achieve this, we gathered a large sample of N = 5314 participants and administered a comprehensive set of questionnaires. These included a Fear of Missing Out (FoMO) scale, which assessed both trait and state aspects of FoMO, the Social Networking Sites-Addiction Test (SNS-AT), designed to gauge tendencies towards SNUD, and the Cognitive Failure Questionnaire (CFQ), which measured everyday cognitive lapses.

**Results:**

Our findings revealed that among non-users of social media, both FoMO and everyday cognitive failures were at their lowest levels. Further, in the group of social media users, we observed a significant relationship between FoMO and cognitive failures, which was mediated by SNUD tendencies. This mediation was particularly pronounced for the state component of FoMO, which encompasses maladaptive thoughts related to online behavior.

**Conclusions:**

While our study is cross-sectional and thus cannot establish causality, one plausible interpretation of our findings is that higher FoMO tendencies may trigger excessive social media use, which in turn could lead to cognitive failures, possibly due to distraction and reduced attention to everyday tasks.

**Supplementary Information:**

The online version contains supplementary material available at 10.1186/s12888-023-05371-x.

## Background

In 2023, it’s estimated that nearly five billion people worldwide will be using social media, underscoring its global significance. Social media platforms hold great appeal for users due to their capacity to facilitate the establishment of social connections and the cultivation of social capital [1]. However, beyond the advantages of social media, there is an ongoing debate surrounding the platforms’ business model that relies on user data as a form of payment and the attention economy. This model has raised concerns about adverse consequences, including the potential for increased time spent online, which may lead to excessive use [2] (sometimes referred to as “social media addiction”), privacy breaches, and the dissemination of misinformation campaigns [3].

The focus of our present study centers on the issue of excessive social media. Within the scientific community, there is an active discourse regarding the precise nature of excessive social media consumption. One aspect under discussion is whether overuse of social media should be classified as an addictive behavior. This debate remains unsettled at this time [4, 5]. In alignment with the nomenclature established in the context of Gaming Disorder, which describes addictive behaviors related to video gaming in the ICD-11 [6], our study employs the term “social networks use disorder” (SNUD) to characterize excessive social media use [7, 8]. Others in the field may currently prefer the term “problematic social media use”, for further discussions around labeling see a work by Elhai, Yang and Levine [9]. It’s important to note that in our study, we prefer the term SNUD tendencies. This said, we conducted our research using a subclinical sample and emphasize that we do not intend to pathologize everyday behavior by employing the term SNUD [10].

In understanding the progression towards SNUD, the I-PACE model proves to be a valuable framework [11]. This model illustrates that Internet Use Disorders, including SNUD, result from the interaction of person, affect, cognition, and execution variables. One crucial variable shedding light on SNUD is the Fear of Missing Out, commonly abbreviated as FoMO [12]. From our view FoMO could be seen as a cognitive, but also affective state wherein an individual fears that others are having rewarding experiences in their absence; but see this work [13]. It is worth noting that FoMO exhibits correlations with traits such as neuroticism and low conscientiousness [14], blurring the line between a trait-like dimension and a state. Hence, depending on the perspective, FoMO could be seen as a trait or state. In this context, Wegmann et al. offer an intriguing perspective: They have developed a modified FoMO scale that provides insights into both trait FoMO and state FoMO [15]. Here, ‘trait’ refers to experiencing FoMO in general, not limited to online environments like social media. Conversely, the items designed to assess state FoMO specifically address the FoMO in online realms, such as constantly being online to avoid missing something. Understanding to what extent trait and state FoMO are differently related to the present variables of interest (SNUD and Cognitive Failure Questionnaire - CFQ; see below) will help other researchers to understand what FoMO variables to best choose in their studies. In our current study, we theoretically position the FoMO trait/state scale within the ‘P-variable’ of the I-PACE model. As mentioned above, the P-variable stands for “person” and comprises among others personality traits.

In addition to assessing FoMO, we also examined individual differences in everyday cognitive failure in the present study [16]. Cognitive failures refer to minor lapses in thought and action, such as forgetting appointments, overlooking information, or accidentally knocking things over [17]. These lapses are a natural consequence of fluctuations in various cognitive domains, including attention, memory, and action control [18]. The susceptibility to cognitive failure derives from a blend of both stable personality factors and situational elements [19]. The latter may encompass conditions such as sleep deprivation, stress, boredom, and information overload [20–23]. Recent research has also indicated that excessive use of social media could potentially instigate cognitive failure [24]. This observation concurs with the hypothesis that the incessant distractions emanating from smartphones and social media platforms might contribute to decreased productivity [25, 26]. Consistent with this, a recent study utilizing a within-study design found that smartphone-checking behavior was correlated with a higher degree of cognitive failure [27]. It’s worth noting that even inverse associations were observed in this study when examining the screen-time measure for social media and other tool applications. This underscores the complexity of the relationships between objective smartphone usage measures and cognitive failure.

Returning to the topic of interruptions resulting from technology use within the context of investigating cognitive failure: Frequent interruptions triggered by incoming push notifications, which can also incite FoMO [28], could likewise contribute to cognitive lapses and reduced productivity. Our anticipation was that higher levels of FoMO, particularly online FoMO (referred to as state FoMO), would correlate with an increased occurrence of cognitive failures. We hypothesized that this relationship would be mediated by tendencies towards SNUD. Put differently, we expected that heightened levels of (state) FoMO might lead to elevated SNUD tendencies, which could, in turn, result in more frequent cognitive failures. This hypothesis is in line with prior research examining the connection between SNUD tendencies and cognitive failure [29]. It’s important to mention that another study also supports the exploration of the link between SNUD and cognitive failure. Hadlington [30] already observed that excessive mobile phone use, which is correlated with SNUD tendencies [31], was positively associated with self-reported cognitive failure. See also another study linking mobile phone addiction to cognitive failures [32].

## Methods

We recruited a total of 5,530 participants through an online website, with the primary aim of investigating the relationships between cognitive failures, FoMO, and SNUD tendencies (other research questions from this data set will be investigated in the future; such as on TikTok Use Disorder and personality)^1^. The study was promoted through a series of media appearances, including print and radio, with a specific emphasis on its investigation of everyday cognitive failures. In the context of this study, participants completed questionnaires designed to assess their levels of FoMO, SNUD tendencies, and everyday cognitive failure. Detailed descriptions of these questionnaires will be provided in the following sections. As a token of appreciation for their participation, participants received insights into their own cognitive failure scores compared to those of other participants. The study was approved by the local ethics committee at Humboldt University in Berlin, Germany.

### Data cleaning

From the initial N = 5,530 participants, we excluded a total of n = 25 individuals who identified as a third gender, as their numbers were insufficient for meaningful statistical analysis. Additionally, n = 3 participants were excluded because their questionnaire responses showed no variance. Moreover, n = 11 participants who were under 18 years old (the study only foresaw to include persons of 18 years and older), and n = 4 participants whose reported age fell outside of the defined upper age cutoff (set at 1.5 times the interquartile range over the third quartile of the age distribution) were also excluded from the study. Furthermore, due to the later introduction of the Fear of Missing Out (FoMO) scale, n = 173 participants were excluded because they did not provide FoMO scores. The final sample comprised n = 5314 participants (1801 males, 3513 females; mean-age: 53.43, SD = 14.84, range 18–94 years). The wider age range is attributable to media outlets where interviews were conducted. Please note that the entire study was conducted in the German language. Regrettably, we did not inquire about participants’ proficiency in the German language, and as a result, we were unable to screen out individuals who may have had difficulty comprehending the items in the online survey. However, we would not assume that participants navigated through the study with the sole intent of receiving feedback on their scores without understanding the content.

### Questionnaires

#### Cognitive failure questionnaire

Participants completed the Cognitive Failure Questionnaire (CFQ) first [16]. The CFQ, in its German version, consists of 32 items [33]. Participants rated the frequency with which various cognitive failures occurred to them in the past six months on a scale ranging from 0 (never) to 4 (very often). The questionnaire demonstrated excellent internal consistencies (α = 0.924, ω = 0.926), with higher scores indicating greater cognitive failure tendencies.

#### FoMO scale

Next, participants filled in Wegmann’s FoMO scale [15], which includes both trait and state facets of Fear of Missing Out (FoMO). This German version of the scale is an adaptation of the original FoMO scale [13]. Wegmann’s FoMO scale comprises twelve items, with five items assessing trait FoMO and seven items examining state FoMO. Participants provided their responses on a five-point Likert scale, ranging from 1 (strongly disagree) to 5 (strongly agree). The internal consistencies for this scale were good (α = 0.788 and ω = 0.810 for trait FoMO; and α = 0.779 and ω = 0.792 for state FoMO), and higher scores indicated greater trait or state FoMO.

#### Social networking sites-addiction test

Finally, participants completed a modified version of the Bergen Facebook Addiction Scale (BFAS)/Bergen Social Media Addiction Scale (BSMAS) [34, 35] as presented in Montag et al. [36] called Social Networking Sites-Addiction Test (SNS-AT): Unlike the BFAS, this version focuses on general social media overuse (not limited to Facebook, as with the BSMAS further developed from the BFAS [35]) and formulates items in the first person perspective (which is different than the BSMAS). The scale consists of six items, and participants rated them on a five-point Likert scale, ranging from 1 (strongly disagree) to 5 (strongly agree). The internal consistencies for this scale were also excellent (α = 0.864, ω = 0.868), with higher scores indicating greater tendencies toward SNUD.

Additionally, all participants were further asked if they used social media or not.

### Statistical analyses

Data cleaning and visualization was performed in MATLAB (v2022b). Statistical analyses were computed with the Jamovi package 2.3.18.0. Participants were categorized into one of three groups based on their social media usage patterns. The first group comprised all participants who indicated that they were active social media users (this means they stated to use social media). The second group consisted of participants who declared that they did not use social media and also exhibited no SNUD tendencies, as evidenced by obtaining the minimum score on the SNS-AT. The third group comprised all participants who stated that they did not use social media but reported SNUD tendencies on the SNS-AT (scores > 6). This peculiar combination of not using social media while still displaying tendencies toward problematic use could imply temporary abstinence or an intentional avoidance of social media due to perceived negative consequences. However, it’s also plausible that individuals in this group provided inconsistent responses for other reasons. Regrettably, we did not include items about past social media usage, making it impossible to verify the status of “ex-users.“ Despite this limitation, we chose to compare this group with the other two for exploratory purposes, but we advise interpreting the results with appropriate caution.

Descriptive statistics for FoMO, SNUD tendencies, and cognitive failure in the three groups are presented in the main text, detailed descriptive statistics for male and female subsamples are given in the supplementary material. The three groups were contrasted by MANOVA with the FoMO, SNS-AT and CFQ as dependent variables and gender and ages as covariates. This was done due to varying age and gender ratios in the three groups (see also supplementary material).

To test our hypothesis regarding the potential mediation of the relationship between FoMO and cognitive failure via SNUD tendencies, we followed a structured approach. Initially, we examined the pairwise relationships between the variables using linear correlation analyses. Subsequently, we employed Jamovi’s advanced mediation model module to construct mediation models. In these models, we designated FoMO as the predictor, SNUD tendencies as the mediating variable, and cognitive failure as the outcome variable. This terminology aligns with mediation model conventions and should not be interpreted as implying causality. We conducted separate mediation models for state and trait FoMO.

### Open science and transparency statement

We collected additional measures from the participants for other research questions. The data linked to the present report are available on the Open Science Framework, together with the analysis code for data cleaning (https://osf.io/bd9y4/).

## Results

**Fig. 1:**
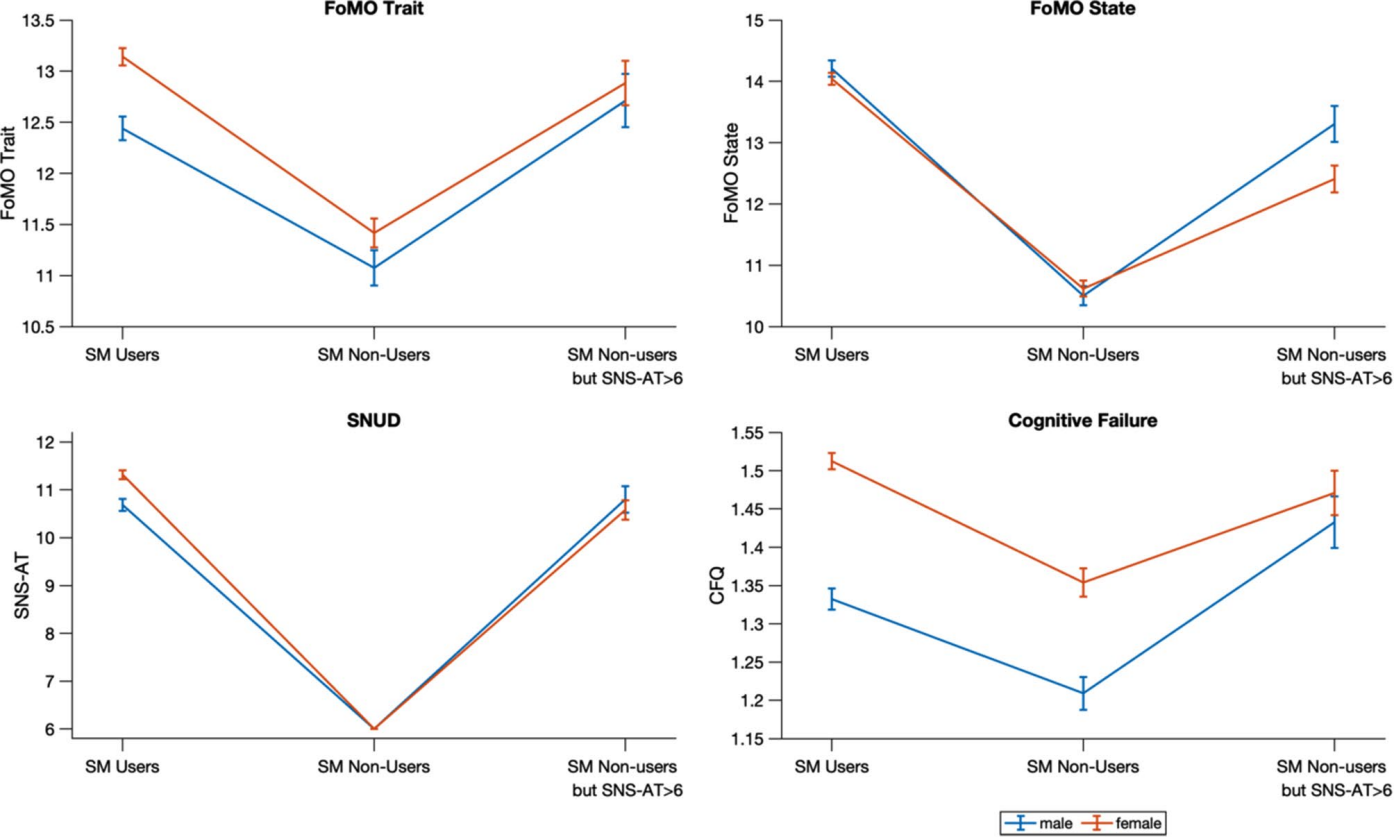
Descriptive statistics (means and standard errors) for trait and state FoMO, SNUD tendencies, and cognitive failure in the three study groups. Results are plotted for male and female participants separately, as indicated by different colors of the lines

Descriptive statistics are presented in Table 1. The three groups differed in all dependent measures (see Fig. 1; Table 2). Social media users (n = 3618; 1147 males, 2471 females, mean-age: 50,89, SD = 14,81) reported higher state and trait FoMO, more SNUD tendencies on the SNS-AT, and more frequent cognitive failure than non-users of social media who did not report SNUD tendencies (n = 1148; 439 males, 709 females; mean-age: 59,47, SD = 12,75). Non-users of social media who still reported higher SNUD tendencies had also higher scores on all measures compared to the non-user group without SNUD tendencies (n = 548; 215 males, 333 females; mean-age: 57,51, SD = 14,53). The scores in this group resembled the scores of active social media users (see Fig. 1). More detailed descriptive statistics regarding males and females are presented in the Supplementary Table 1.

**Table 1:** Descriptive statistics of the investigated groups (1 = social media users; 2 = social media non-users, 3 = social media non-users, but SNS-AT > 6)

	Group	N	Missing	Mean	Median	SD	Minimum	Maximum
Age	1	3618	0	50.89	53.00	14.808	18	89
	2	1148	0	59.47	61.00	12.748	20	91
	3	548	0	57.51	60.00	14.534	18	94
Trait FoMO	1	3618	0	12.92	12.00	4.132	5	25
	2	1148	0	11.29	11.00	3.714	5	23
	3	548	0	12.82	12.00	3.902	5	25
State FoMO	1	3618	0	14.09	14.00	4.687	7	33
	2	1148	0	10.57	10.00	3.368	7	25
	3	548	0	12.76	12.00	4.143	7	28
SNS-AT (SNUD)	1	3618	0	11.12	10.00	4.535	6	30
	2	1148	0	6.00	6.00	0.000	6	6
	3	548	0	10.67	9.00	3.821	7	30
CFQ (Cognitive Failure Questionnaire)*	1	3618	0	1.46	1.41	0.512	0.156	3.97
	2	1148	0	1.30	1.23	0.481	0.188	3.50
	3	548	0	1.46	1.41	0.515	0.313	3.19

FoMO: Fear of Missing Out, SNS-AT: Social Networking Sites-Addiction Test, SNUD: Social Networks Use Disorder; * please note that no sum scores across items were created here

**Table 2:** Univariate Tests following MANCOVA on group differences between active social media users, non-users of social media without SNUD tendencies, and non-users of social media with SNUD-tendencies, controlling for gender and age differences

	Dependent Variable	Sum of Squares	df	Mean Square	F	*p*
Group	CFQ	22.21	2	11.106	46.892	< 0.001
	SNS-AT (SNUD)	23122.66	2	11561.332	805.725	< 0.001
	State FoMO	10908.00	2	5453.998	286.842	< 0.001
	Trait FoMO	2366.91	2	1183.454	79.193	< 0.001
Gender	CFQ	28.98	1	28.984	122.382	< 0.001
	SNS-AT (SNUD)	181.61	1	181.614	12.657	< 0.001
	State FoMO	39.45	1	39.448	2.075	0.150
	Trait FoMO	373.40	1	373.400	24.987	< 0.001
Group ✻ Gender	CFQ	1.64	2	0.818	3.455	0.032
	SNS-AT (SNUD)	77.79	2	38.894	2.711	0.067
	State FoMO	88.82	2	44.409	2.336	0.097
	Trait FoMO	17.32	2	8.660	0.579	0.560
Age	CFQ	71.75	1	71.747	302.940	< 0.001
	SNS-AT (SNUD)	5963.55	1	5963.554	415.608	< 0.001
	State FoMO	839.13	1	839.133	44.133	< 0.001
	Trait FoMO	6214.92	1	6214.922	415.883	< 0.001
Residuals	CFQ	1256.88	5307	0.237		
	SNS-AT (SNUD)	76150.01	5307	14.349		
	State FoMO	100906.89	5307	19.014		
	Trait FoMO	79307.46	5307	14.944		

CFQ: Cognitive Failure Questionnaire; SNS-AT: Social Networking Sites-Addiction Test; SNUD: Social Networks Use Disorder, FoMO: Fear of Missing Out

Focusing only on the group of active social media users (n = 3618), we observed significant intercorrelations between all study variables (see Table 3). Correlation coefficients between the questionnaire measures indicated moderate effect sizes (exception: CFQ and state FoMO was in a small effect size area). Age was inversely related to all questionnaire variables with small to moderate effect sizes.

**Table 3:** Linear correlations between FoMO Variables, SNUD tendencies (SNS-AT), cognitive failure (CFQ), and age in the active social media user sample (correlations controlling for age can be found in the supplement; see ST4)

		Trait FoMO	State FoMO	CFQ	SNS-AT	Age
Trait FoMO	Pearson’s r	—				
	*p*-value	—				
State FoMO	Pearson’s r	0.375	—			
	*p*-value	< 0.001	—			
CFQ (Cognitive Failure Questionnaire)	Pearson’s r	0.368	0.192	—		
	*p*-value	< 0.001	< 0.001	—		
SNS-AT (SNUD)	Pearson’s r	0.418	0.516	0.354	—	
	*p*-value	< 0.001	< 0.001	< 0.001	—	
Age	Pearson’s r	-0.309	-0.148	-0.254	-0.335	—
	*p*-value	< 0.001	< 0.001	< 0.001	< 0.001	—

FoMO: Fear of Missing Out; SNS-AT: Social Networking Sites-Addiction Test; SNUD: Social Networks Use Disorder

**Fig. 2:**
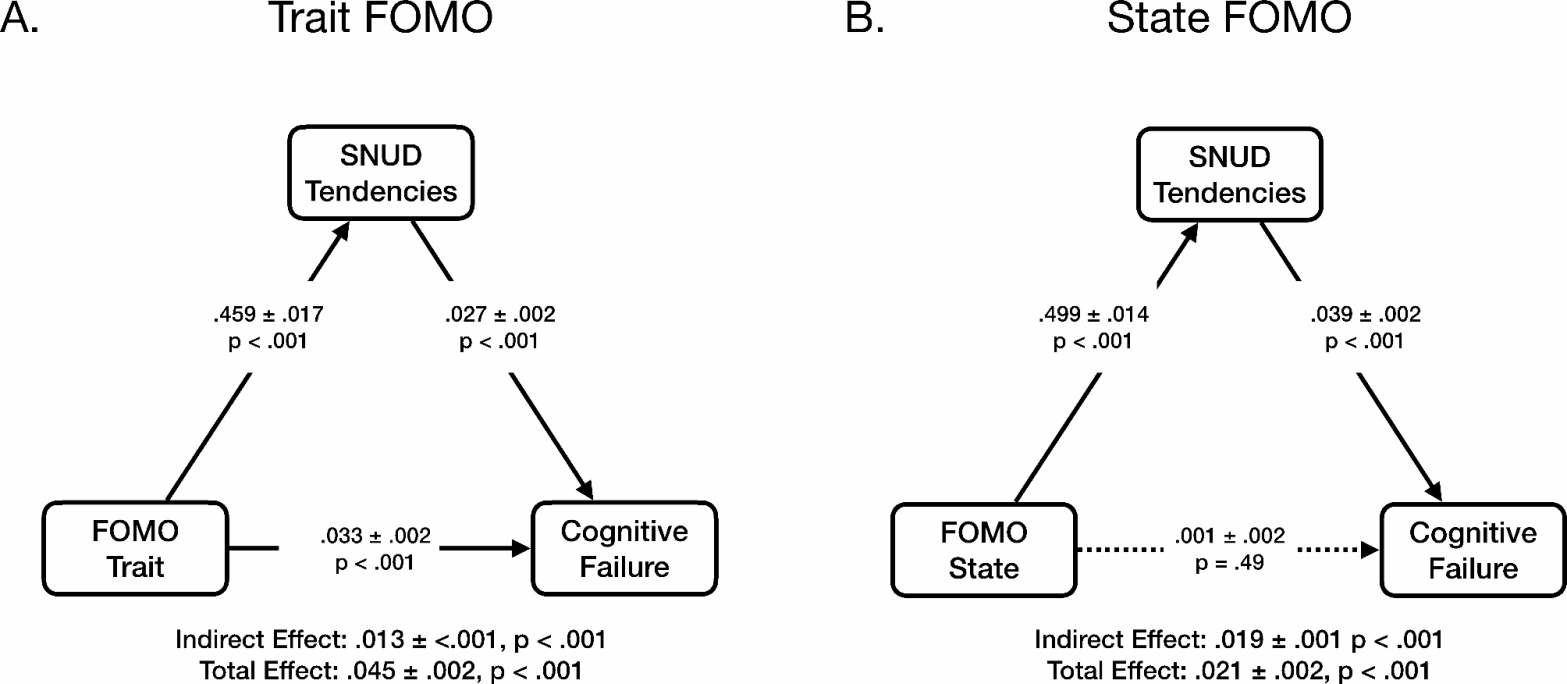
Mediation models for trait FoMO (left side: **A**) and state FoMO (right side: **B**). Effect estimates with standard errors are given for the indirect effect, its two components, the direct effect, and the total effect

The subsequent mediation analysis revealed a complete mediation of the relationship between state FoMO and cognitive failure through SNUD tendencies and a partial mediation of the relationship between trait FoMO and cognitive failure through SNUD tendencies (see Fig. 2). Adding age and gender as additional factors to the model did not change these mediations in a meaningful way (see supplementary materials).

## Discussion

The primary objective of this study was to explore the intricate relationships between FoMO, SNUD tendencies, and cognitive failures. Our findings bring these associations to the forefront, providing insight into the complex interconnections between these factors. Significantly, this study offers a comprehensive examination, encompassing both trait and state FoMO, SNUD tendencies, and cognitive failures as assessed by the Cognitive Failure Questionnaire (CFQ) within a single investigation.

In the group of active social media users, we identified a network of positive associations among all three variables. As depicted in Table 3 the overall correlations between FoMO, SNUD and CFQ are in the moderate effect size area (with the exception for the FoMO state-CFQ association, which falls in the small effect size area). Therefore, one can assume robust associations between the variables, at least in the active social media user group. These findings align with prior research, which consistently shows robust connections between FoMO and elevated SNUD tendencies [12]. This further underscores the notion that the FoMO on online experiences is closely linked to the inclination toward problematic social media use (or SNUD tendencies). The present study also highlights the importance of differentiating between the trait and state facets of FoMO variables. To elaborate, we found that the trait FoMO variable exhibited a stronger association with CFQ than the state FoMO variable did. Conversely, when examining FoMO and SNUD tendencies, we observed the opposite pattern. Notably, state FoMO (specifically, online FoMO) showed a stronger association with SNUD tendencies than the trait FoMO did. This distinction arises because the trait FoMO items capture a more general sense of FoMO without explicitly referencing the online context in their respective items.

The examination of connections between SNUD tendencies and cognitive failures remains a relatively uncharted area. However, our findings are consistent with emerging evidence. For instance, a study observed associations between performance indices on cognitive tasks, such as the Wisconsin Card Sorting Test, and scores on the Bergen Social Media Addiction Scale (BSMAS) in problematic social media users [37]. Similarly, other studies have linked “dependence on SNS” (p. 121) and more cognitive failures [29] with consistent findings reported in a bit older work dealing with problematic mobile phone use and cognitive failure [30], see also a more recent study [38]. Niu et al. also noted that smartphone presence can adversely affect cognitive functions, with FoMO serving as a moderating variable [39].

To the best of our knowledge, our study stands as one of the first to comprehensively investigate FoMO (including both trait and state scales), SNUD tendencies, and cognitive failure (as assessed by the CFQ) within a single study. Our results corroborate the hypothesized positive associations between FoMO and cognitive failure, with SNUD tendencies emerging as a mediator in this relationship. Specifically, we found that SNUD tendencies fully mediated the relationship between state FoMO and cognitive failure, while the relationship between trait FoMO and cognitive failure exhibited a partial mediation through SNUD tendencies. The slight differences in the mediation models likely can be explained by the different strength of associations as mentioned earlier in the discussion.

While our findings provide valuable insights, several limitations warrant consideration. Firstly, our study’s cross-sectional nature precludes any inference of causality. In principle, it is also imaginable that persons scoring higher on cognitive failure are more prone to experience FoMO and SNUD. Perhaps persons with more cognitive failure also have more problems in self-regulation and develop more easily SNUD tendencies. To establish causal relationships, future experimental research is needed, exploring diverse variables within our mediation model. Secondly, self-report measures inherently carry the potential for biases, including social desirability and a lack of introspection among some participants. Additionally, our non-representative sample limits the generalizability of our findings, although they are broadly consistent with existing literature. Finally, other variables are of interest to be studied in this context of the present research question including personality traits or other sociodemographic variables. This would make an interesting research endeavor.

## Conclusions

Despite these limitations, our study underscores the robust associations among FoMO, SNUD tendencies, and cognitive failure. The possibility that excessive social media use may lead to cognitive failures highlights the importance of designing healthier social media platforms. Platforms that focus on user well-being rather than exploiting addictive design elements can contribute to a more sustainable and responsible digital landscape [40, 41]. Such efforts may ultimately require a shift away from data-driven business models, encouraging exploration of alternative payment structures in the world of social media [42].

In conclusions, our study contributes to the growing body of knowledge about the intricate relationships between FoMO, SNUD tendencies, and cognitive failures. It underscores the need for further research and proactive measures to promote healthier and more mindful engagement with social media platforms in our digitally connected world.

### Electronic supplementary material

Below is the link to the electronic supplementary material.

Supplementary Material 1

## Data Availability

The data is available via this link at the Open Science Framework: https://osf.io/bd9y4/.

## Footnotes

1 Of note, only few participants from the present sample stated to use TikTok. Therefore, this data cannot be presented here and the data collection in this regard is ongoing. Please note that the study was not framed to investigate TikTok use and so no bias in data gathering in this regard could have happened.
