# Supplementary material for: Social media use and everyday cognitive failure: investigating the fear of missing out and social networks use disorder relationship
Source: BMC Psychiatry. 2023 Nov 24;23:872. doi: 10.1186/s12888-023-05371-x (PMC10668512; doi:10.1186/s12888-023-05371-x)
Supplement: Supplementary file 1 — Supplementary Material 1 [file 12888_2023_5371_MOESM1_ESM.docx]

**Supplementary Material**

We present additional descriptive statistics in Supplementary Table 1 (ST1), and give detailed information on the mediation models in Supplementary Table 2 (ST2) and 3 (ST3); see also the additional Figure SF1. See for a further correlation Table ST4.

| ST1: Descriptive statistics for males and females in the three investigated groups | | | | | | | | | | | | | | | | | |
| --- | --- | --- | --- | --- | --- | --- | --- | --- | --- | --- | --- | --- | --- | --- | --- | --- | --- |
|  | | **group** | | **gender** | | **N** | | **Mean** | | **Median** | | **SD** | | **Minimum** | | **Maximum** | |
| Trait FoMO |  | Social Media Use: yes |  | m |  | 1147 |  | 12.44 |  | 12 |  | 3.943 |  | 5 |  | 25 |  |
|  |  |  |  | f |  | 2471 |  | 13.14 |  | 13 |  | 4.200 |  | 5 |  | 25 |  |
|  |  | Social Media Use: no and scores SMA = 6 |  | m |  | 439 |  | 11.08 |  | 11 |  | 3.591 |  | 5 |  | 23 |  |
|  |  |  |  | f |  | 709 |  | 11.42 |  | 11 |  | 3.784 |  | 5 |  | 23 |  |
|  |  | Social Media Use: no and scores SNA > 6 |  | m |  | 215 |  | 12.71 |  | 12 |  | 3.818 |  | 5 |  | 24 |  |
|  |  |  |  | f |  | 333 |  | 12.88 |  | 13 |  | 3.959 |  | 5 |  | 25 |  |
| State FoMo |  | Social Media Use: yes |  | m |  | 1147 |  | 14.21 |  | 14 |  | 4.505 |  | 7 |  | 33 |  |
|  |  |  |  | f |  | 2471 |  | 14.04 |  | 13 |  | 4.770 |  | 7 |  | 33 |  |
|  |  | Social Media Use: no and scores SMA = 6 |  | m |  | 439 |  | 10.50 |  | 10 |  | 3.226 |  | 7 |  | 23 |  |
|  |  |  |  | f |  | 709 |  | 10.62 |  | 10 |  | 3.455 |  | 7 |  | 25 |  |
|  |  | Social Media Use: no and scores SNA > 6 |  | m |  | 215 |  | 13.31 |  | 13 |  | 4.309 |  | 7 |  | 27 |  |
|  |  |  |  | f |  | 333 |  | 12.41 |  | 12 |  | 3.999 |  | 7 |  | 28 |  |
| SNUD |  | Social Media Use: yes |  | m |  | 1147 |  | 10.69 |  | 10 |  | 4.264 |  | 6 |  | 30 |  |
|  |  |  |  | f |  | 2471 |  | 11.32 |  | 11 |  | 4.642 |  | 6 |  | 30 |  |
|  |  | Social Media Use: no and scores BMSAS = 6 |  | m |  | 439 |  | 6.00 |  | 6 |  | 0.000 |  | 6 |  | 6 |  |
|  |  |  |  | f |  | 709 |  | 6.00 |  | 6 |  | 0.000 |  | 6 |  | 6 |  |
|  |  | Social Media Use: no and scores BMSAS > 6 |  | m |  | 215 |  | 10.80 |  | 10 |  | 4.018 |  | 7 |  | 30 |  |
|  |  |  |  | f |  | 333 |  | 10.58 |  | 9 |  | 3.693 |  | 7 |  | 24 |  |
| CFQ |  | Social Media Use: yes |  | m |  | 1147 |  | 1.33 |  | 1.28 |  | 0.470 |  | 0.156 |  | 3.22 |  |
|  |  |  |  | f |  | 2471 |  | 1.51 |  | 1.47 |  | 0.521 |  | 0.156 |  | 3.97 |  |
|  |  | Social Media Use: no and scores BMSAS = 6 |  | m |  | 439 |  | 1.21 |  | 1.16 |  | 0.451 |  | 0.188 |  | 2.84 |  |
|  |  |  |  | f |  | 709 |  | 1.35 |  | 1.31 |  | 0.491 |  | 0.188 |  | 3.50 |  |
|  |  | Social Media Use: no and scores BMSAS > 6 |  | m |  | 215 |  | 1.43 |  | 1.47 |  | 0.493 |  | 0.313 |  | 3.03 |  |
|  |  |  |  | f |  | 333 |  | 1.47 |  | 1.38 |  | 0.529 |  | 0.313 |  | 3.19 |  |
|  | | | | | | | | | | | | | | | | | |

SF 1: Mediation model with the predictor FOMO, SNUD tendencies as mediator, cognitive failure as dependent measure, and age and gender as additional covariates. The same model was fitted for state and trait FOMO, see ST2 and ST3.

| ST2: Mediation Model for State FoMO with Gender and Age as additional covariates. Shown are all indirect and total effects | | | | | | | | | | | | | | | | | |
| --- | --- | --- | --- | --- | --- | --- | --- | --- | --- | --- | --- | --- | --- | --- | --- | --- | --- |
|  | | | | | | | | **95% C.I. (a)** | | | |  | | | | | |
| **Type** | | **Effect** | | **Estimate** | | **SE** | | **Lower** | | **Upper** | | **β** | | **z** | | **p** | |
| Indirect |  | Gender ⇒ SNUD ⇒ CFQ |  | 0.01644 |  | 0.00437 |  | 0.00788 |  | 0.02499 |  | 0.0149 |  | 3.76 |  | < .001 |  |
|  |  | FoMO state ⇒ SNUD⇒ CFQ |  | 0.01478 |  | 0.00106 |  | 0.01269 |  | 0.01686 |  | 0.1352 |  | 13.90 |  | < .001 |  |
|  |  | Age ⇒ SNUD⇒ CFQ |  | -0.00255 |  | 2.15e-4 |  | -0.00297 |  | -0.00212 |  | -0.0736 |  | -11.84 |  | < .001 |  |
| Component |  | Gender ⇒ SNUD |  | 0.51435 |  | 0.13234 |  | 0.25498 |  | 0.77373 |  | 0.0528 |  | 3.89 |  | < .001 |  |
|  |  | SNUD ⇒ CFQ |  | 0.03195 |  | 0.00211 |  | 0.02782 |  | 0.03608 |  | 0.2829 |  | 15.16 |  | < .001 |  |
|  |  | FoMO state ⇒ SNUD |  | 0.46242 |  | 0.01325 |  | 0.43645 |  | 0.48838 |  | 0.4780 |  | 34.91 |  | < .001 |  |
|  |  | Age ⇒ SNUD |  | -0.07968 |  | 0.00421 |  | -0.08792 |  | -0.07144 |  | -0.2602 |  | -18.95 |  | < .001 |  |
| Direct |  | Gender ⇒ CFQ |  | 0.14843 |  | 0.01682 |  | 0.11547 |  | 0.18139 |  | 0.1349 |  | 8.83 |  | < .001 |  |
|  |  | FoMO state ⇒ CFQ |  | 0.00290 |  | 0.00194 |  | -9.09e−4 |  | 0.00670 |  | 0.0265 |  | 1.49 |  | 0.136 |  |
|  |  | Age ⇒ CFQ |  | -0.00502 |  | 5.59e-4 |  | -0.00611 |  | -0.00392 |  | -0.1450 |  | -8.97 |  | < .001 |  |
| Total |  | Gender ⇒ CFQ |  | 0.16487 |  | 0.01731 |  | 0.13095 |  | 0.19879 |  | 0.1498 |  | 9.53 |  | < .001 |  |
|  |  | FoMO ⇒ CFQ |  | 0.01767 |  | 0.00173 |  | 0.01428 |  | 0.02107 |  | 0.1618 |  | 10.20 |  | < .001 |  |
|  |  | Age ⇒ CFQ |  | -0.00756 |  | 5.50e-4 |  | -0.00864 |  | -0.00648 |  | -0.2186 |  | -13.75 |  | < .001 |  |
| Note. Confidence intervals computed with method: Standard (Delta method) | | | | | | | | | | | | | | | | | |
| Note. Betas are completely standardized effect sizes | | | | | | | | | | | | | | | | | |
|  | | | | | | | | | | | | | | | | | |

| ST3: Mediation Model for Trait FoMO with Gender and Age as additional covariates. Shown are all indirect and total effects | | | | | | | | | | | | | | | | | |
| --- | --- | --- | --- | --- | --- | --- | --- | --- | --- | --- | --- | --- | --- | --- | --- | --- | --- |
|  | | | | | | | | **95% C.I. (a)** | | | |  | | | | | |
| **Type** | | **Effect** | | **Estimate** | | **SE** | | **Lower** | | **Upper** | | **β** | | **z** | | **p** | |
| Indirect |  | Gender ⇒ SNUD⇒ CFQ |  | 0.00471 |  | 0.00348 |  | -0.00211 |  | 0.01152 |  | 0.00428 |  | 1.35 |  | 0.176 |  |
|  |  | Age ⇒ SNUD⇒ CFQ |  | -0.00167 |  | 1.74e-4 |  | -0.00201 |  | -0.00133 |  | -0.04827 |  | -9.60 |  | < .001 |  |
|  |  | FoMO trait ⇒ SNUD⇒ CFQ |  | 0.00915 |  | 8.27e-4 |  | 0.00753 |  | 0.01077 |  | 0.07381 |  | 11.06 |  | < .001 |  |
| Component |  | Gender1 ⇒ SNUD |  | 0.19547 |  | 0.14357 |  | -0.08593 |  | 0.47687 |  | 0.02006 |  | 1.36 |  | 0.173 |  |
|  |  | SNUD⇒ CFQ |  | 0.02408 |  | 0.00189 |  | 0.02037 |  | 0.02779 |  | 0.21322 |  | 12.72 |  | < .001 |  |
|  |  | Age ⇒ SNUD |  | -0.06933 |  | 0.00473 |  | -0.07860 |  | -0.06005 |  | -0.22637 |  | -14.66 |  | < .001 |  |
|  |  | FoMO trait ⇒ SNUD |  | 0.37987 |  | 0.01696 |  | 0.34664 |  | 0.41310 |  | 0.34615 |  | 22.40 |  | < .001 |  |
| Direct |  | Gender ⇒ CFQ |  | 0.13597 |  | 0.01636 |  | 0.10391 |  | 0.16803 |  | 0.12356 |  | 8.31 |  | < .001 |  |
|  |  | Age ⇒ CFQ |  | -0.00344 |  | 5.55e-4 |  | -0.00453 |  | -0.00236 |  | -0.09957 |  | -6.21 |  | < .001 |  |
|  |  | FoMO trait ⇒ CFQ |  | 0.02947 |  | 0.00206 |  | 0.02543 |  | 0.03351 |  | 0.23783 |  | 14.30 |  | < .001 |  |
| Total |  | Gender ⇒ CFQ |  | 0.14068 |  | 0.01672 |  | 0.10792 |  | 0.17344 |  | 0.12784 |  | 8.42 |  | < .001 |  |
|  |  | Age ⇒ CFQ |  | -0.00511 |  | 5.51e-4 |  | -0.00619 |  | -0.00403 |  | -0.14784 |  | -9.28 |  | < .001 |  |
|  |  | FoMO trait ⇒ CFQ |  | 0.03862 |  | 0.00197 |  | 0.03475 |  | 0.04249 |  | 0.31163 |  | 19.56 |  | < .001 |  |
| Note. Confidence intervals computed with method: Standard (Delta method) | | | | | | | | | | | | | | | | | |
| Note. Betas are completely standardized effect sizes | | | | | | | | | | | | | | | | | |
|  | | | | | | | | | | | | | | | | | |

| ST4: Correlations between the study variables controlling for age | | | | | | | | | | | |
| --- | --- | --- | --- | --- | --- | --- | --- | --- | --- | --- | --- |
|  | |  | | **Trait FoMO** | | **State FoMO** | | **CFQ** | | **SNS-AT** | |
| Trait FoMO |  | Pearson's r |  | — |  |  |  |  |  |  |  |
|  |  | p-value |  | — |  |  |  |  |  |  |  |
| State FoMO |  | Pearson's r |  | 0.350 |  | — |  |  |  |  |  |
|  |  | p-value |  | < .001 |  | — |  |  |  |  |  |
| CFQ |  | Pearson's r |  | 0.314 |  | 0.161 |  | — |  |  |  |
|  |  | p-value |  | < .001 |  | < .001 |  | — |  |  |  |
| SNS-AT (SNUD) |  | Pearson's r |  | 0.351 |  | 0.500 |  | 0.295 |  | — |  |
|  |  | p-value |  | < .001 |  | < .001 |  | < .001 |  | — |  |
| Note. controlling for 'age'.  FoMO: Fear of Missing Out, CFQ: Cognitive Failure Questionnaire, SNS-AT: Social Networking Sites-Addiction Test, SNUD: Social Networks Use Disorder | | | | | | | | | | | |
|  | | | | | | | | | | | |
